# Measuring Dynamic Tendon Torsion Using Ultrasound Speckle Tracking: Validation with Silicone Phantom and In Vivo Application on Human Tibialis Posterior Tendon

**DOI:** 10.3390/s26041187

**Published:** 2026-02-11

**Authors:** Kun-Lin Hung, De-Kai Syu, Wei-Ning Lee, Pei-Yu Chen, Chen-Chie Wang, Wen-Siang Chen, Che-Yu Lin, Hsing-Kuo Wang

**Affiliations:** 1 School and Graduate Institute of Physical Therapy, College of Medicine, National Taiwan University, Taipei City 100025, Taiwan; f08428001@ntu.edu.tw; 2 Department of Orthopedics, Fu Jen Catholic University Hospital, New Taipei City 243089, Taiwan; a00416@mail.fjuh.fju.edu.tw; 3 Biomedical Engineering Programme, The University of Hong Kong, Pokfulam, Hong Kong; wnlee@eee.hku.hk; 4 Department of Orthopedic Surgery, National Taiwan University Hospital, Taipei City 100225, Taiwan; chenpeiyu@ntu.edu.tw; 5 Department of Orthopedics Surgery, Taipei Tzu Chi Hospital, Buddhist Tzu Chi Medical Foundation, New Taipei City 231016, Taiwan; xavier-wang@yahoo.com.tw; 6 Department of Orthopedics, School of Medicine, Tzu Chi University, Hualien County 970374, Taiwan; 7 Department of Physical Medicine and Rehabilitation, National Taiwan University Hospital, Taipei City 100229, Taiwan; wenshiangchen@ntu.edu.tw; 8 Institute of Applied Mechanics, College of Engineering, National Taiwan University, Taipei City 106319, Taiwan; cheyu@ntu.edu.tw; 9 Center of Physical Therapy, National Taiwan University Hospital, Taipei City 100229, Taiwan

**Keywords:** ultrasonography, speckle tracking, tendon torsion, validity, reliability, silicone phantom, tibialis posterior tendon

## Abstract

**Highlights:**

**What are the main findings?**

- Transverse plane speckle tracking is validated for quantifying dynamic torsion angles on silicone phantoms.
- Speckle tracking can be applied to measure dynamic torsion of human tibialis posterior tendon with reliable results.

**What are the implications of the main findings?**

- Transverse plane speckle tracking is a valid and reliable method for assessing tendons’ dynamic torsion characteristics.
- Dynamic torsion assessments via speckle tracking may provide functionally and clinically relevant information regarding tendons’ dynamic torsion characteristics.

**Abstract:**

The torsional characteristics of human tendons are recognized to have functional and clinical relevance, but are underexplored due to the limited in vivo assessment methods available to measure the dynamic torsion characteristics of a tendon during movement. This study aimed to validate the use of transverse plane ultrasound speckle tracking (ST) for measuring dynamic torsion on silicone phantoms, and to evaluate the capability and reliability of ST in measuring dynamic torsion of the human tibialis posterior tendon (TPT) in vivo. Of the ten silicone phantoms tested in the validation study, ST measurement results strongly correlated with the referencing marker tracking method (*R*^2^ = 0.81–0.95) and had measurement error similar to or smaller than the hypothesized accuracy of 3° (*p* > 0.045). Subsequently, when ST was applied to nineteen healthy participants’ TPT in vivo, it was capable of characterizing the dynamic external torsion of the TPT during 0–20° passive foot pronation. Strong correlations were found between the ST-measured angle and the foot pronation angle (*R*^2^ = 0.98–0.99), and the test–retest reliability was moderate to good (ICC = 0.73–0.87). These findings suggested that ST is a valid and reliable method for measuring dynamic tendon torsion characteristics.

## 1. Introduction

Tendon torsion refers to a structural twist along the long axis of a tendon and has been reported in the posterior tibial, Achilles, peroneus, and extensor carpi ulnaris tendons [1,2,3,4,5]. Several functional implications of tendon torsion have been proposed, including resistance against rotational or multidirectional forces [6], equalization of intratendinous excursion discrepancies caused by the curved anatomical course [7], facilitation of energy-storage and release mechanisms of the tendon [8,9,10], modulation of intratendinous pressure [11], and alteration in mechanical strength [12,13,14,15]. Clinical implications of tendon torsion have also been examined in a small number of experimental studies. Roukis et al. found that the absence of tendon torsion at rest can induce intratendinous excursion discrepancies in the tibialis posterior tendon (TPT) when the foot is moved passively into pronation and supination, which may cause an unnatural shearing within the tendon structure [7]. Pringles et al. discovered that Achilles tendons with type II torsion exhibited greater intratendinous pressures during straight-knee calf stretch and eccentric ankle dorsiflexion compared to less torsional type I Achilles tendons [11]. This finding suggests that a higher degree of anatomical tendon torsion in the Achilles may lead to increased intratendinous pressures during stretch or eccentric contractions; this may increase the risk of tendinopathy through the compression of vascular supply to the tendon [5,10]. Despite evidence suggesting degrees of tendon torsion at rest may have functional and clinical significance, little is known about the extent of dynamic torsion changes under movement or mechanical load.

The dynamic changes in tendon torsion (or “dynamic torsion”) refer to the twisting motion of a tendon under torsional loading, resulting in rotation of the tendon’s cross-section on the transverse plane. To evaluate tendons’ dynamic torsion characteristics during movement, studies have used ultrasound imaging to capture the rotation of tendons’ cross-sections in the transverse plane due to its non-invasiveness, high-resolution, and real-time characteristics, as well as its ability to reliably capture the musculotendinous tissue morphology and architecture [16,17,18]. Obst et al. [17] used grayscale imaging to assess the dynamic torsion of the Achilles tendon by measuring the orientational changes (in degrees) of the principal axes of the tendon’s cross-section and observed the accentuation of torsion around the tendon midportion during isometric plantar flexion. Wang et al. [18] used vector Doppler imaging to assess the rotational velocities of cross-sections of the flexor digitorum tendons and observed tendon rotation during finger flexion. Although both methods allowed monitoring of the changes in tendon torsion during dynamic tasks, the grayscale imaging method may not be able to identify dynamic tendon torsion angles if the principal axes are difficult to define (i.e., in round-shaped tendons). Meanwhile, vector Doppler imaging cannot provide quantified measures of dynamic torsion angles and may be limited in its applicability due to inherent measuring angle dependency [19].

Ultrasound speckle tracking (ST) could be a solution for quantifying dynamic torsion angles. Transverse plane ST has been used in echocardiography to quantify two-dimensional local tissue displacements [19] and to assess the twisting and untwisting (torsional) motions of the left ventricle [20,21,22]. Compared to Doppler imaging, ST measurement results are not affected by the probe angle and have superior spatial resolution and lower noise sensitivity [19,23]. For in vivo measurements of tendon movements, ST has been used to assess longitudinal displacements of the flexor digitorum [24,25], posterior tibial [26], Achilles [27,28,29,30], and patellar tendons [31,32]; a measurement precision of 0.3 mm was reported for the flexor digitorum superficialis tendon [24,25]. Although ST has not yet been used to measure transverse plane tendon movements, given the sub-millimeter accuracy of longitudinal displacement estimations [24,25,33] and the suggested better lateral displacement estimation accuracy in transverse plane images of anisotropic tendon tissue [34], this study believe that ST would be capable of accurately measuring local tendon displacements in its transverse plane. Such measurements could be regarded as tangential displacements and used to calculate a tendon’s dynamic torsion angle.

This study aimed to validate the use of ST in measuring dynamic torsion angles and to evaluate its feasibility and reliability for such measurements in vivo. First, a set of cylindrical-shaped silicone phantoms (6 mm in radius) was used to examine the comparability and accuracy of ST against the gold-standard marker tracking (MT) method [24,35]. Then, the validated ST methodology was applied to the human TPT during passive foot pronation motion to assess its feasibility and reliability in measuring in vivo dynamic torsion angles. The TPT was chosen because it exhibits structural torsion, and previous research has suggested that the tendon undergoes dynamic torsion during ankle and foot movements [1]. The results are expected to provide foundational evidence for developing quantitative dynamic torsion assessment methods and for characterizing the TPT’s dynamic torsion characteristics in vivo. These outcomes may be valuable for improving understanding of the pathomechanics of the TPT by observing its dynamic torsion in response to foot movement, and for expanding the scope of clinical ultrasonographic assessment and outcome measurements in managing TPT pathologies (e.g., tendinopathy, overuse injuries, and posterior tibial tendon dysfunction) [1,36,37]. This study expected that ST could yield comparable results to those of the MT method, and the measurement error is expected to be close to 3 degrees in the 6-mm radius phantoms, based on the previously reported measurement error of 0.3 mm in longitudinal flexor digitorum superficialis tendon displacements [24,25]. This study also expected ST to be capable of measuring in vivo dynamic torsion angles of the TPT during passive foot pronation, and the measurements to be reliable.

## 2. Materials and Methods

This study was approved by the institutional review board of National Taiwan University Hospital (Reference No. 202112174RINA) and was conducted according to the ethical standards of the 1964 Declaration of Helsinki. All participants provided written informed consent, and their rights were protected.

### 2.1. Validation of ST Dynamic Torsion Assessment

The validation of ST for measuring dynamic torsion angles was conducted by comparing ST results to those of the MT method. Ten silicon phantoms with a radius of 6 mm and a length of 60 mm were used in the study to simulate the dynamic torsion movement of a tendon on a torsional mechanical testing system (T-MTS) (AD-10TA, Shimadzu Corporation, Tokyo, Japan). The dynamic torsion angles of the phantoms were measured by both ST and MT analyses, and the measurement errors of the ST methods were calculated in reference to the MT results.

#### 2.1.1. Experimental Materials

**Figure 1:**
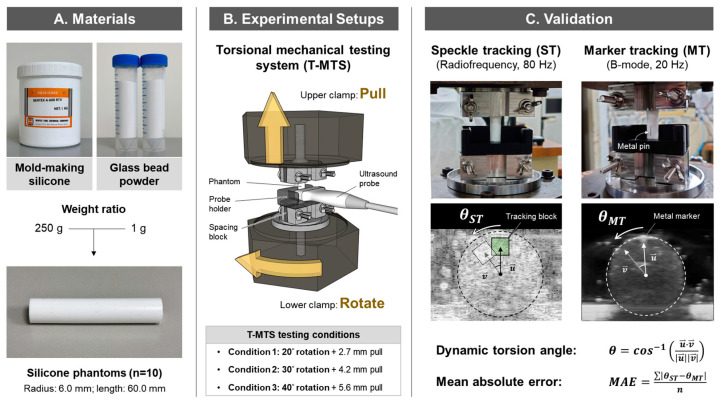
Validation of speckle tracking in measuring dynamic torsion angles. (**A**) Ten cylindrical-shaped silicone phantoms were prepared by mixing mold-making silicone with glass bead powder with a standardized size of 6.0 mm in radius and 60.0 mm in length; (**B**) The silicone phantoms were mounted on a torsional mechanical testing system that rotates and pulls the phantom simultaneously. Three testing conditions (conditions 1, 2, and 3) were used to induce different magnitudes of dynamic torsion in the phantom; (**C**) Speckle tracking and the reference marker tracking methods were conducted separately on the same set of phantoms to assess the mean absolute error of speckle-tracking measurements.

The silicon phantoms were prepared by mixing mold-making silicone rubber (A-600 RTV, Chung-Chan Industrial Chemicals, Taipei, Taiwan) with 40–70 µm glass bead powder (G40-70μ, Shan-Tzauh Enterprise, New Taipei City, Taiwan) at a 250:1 weight ratio. The addition of glass bead powder to the silicone mix was intended to create speckles in the ultrasound images [38], thereby making the phantom images resemble the homogeneous, isotropic tendon images obtained in the transverse plane. The silicone phantoms were then mounted vertically on the T-MTS by two custom-made stainless-steel clamps for dynamic torsion testing (Figure 1A). The vertical alignment of the phantom to the T-MTS was confirmed with a laser level. To control the mechanical testing conditions, the stainless-steel clamps covered 10 mm of phantom length at both ends, leaving 40 mm of phantom in the midportion to be twisted by the T-MTS. A set of 3D-printed spacing blocks was put between the clamps to ensure 50% compression of the phantom at the clamping sites (Figure 1B).

#### 2.1.2. Dynamic Torsion Mechanical Testing

To simulate the suggested dynamic torsion and excursion of the TPT during ankle and foot motions, the T-MTS was programmed to twist and pull the phantom at a ratio of 7.2° to 1 mm, which is close to the ratio of 47.5° TPT structural torsion to the reported 6.8–7.2 mm of displacement in passive foot pronation [1,39,40]. To induce different magnitudes of dynamic torsion of the phantom, three T-MTS testing conditions were set, consisting of 20°, 30°, or 40° rotation at the lower clamp and simultaneous pulls at the upper clamp; these were referred to as conditions 1, 2, and 3 in the study, respectively (Figure 1B). These rotation ranges were selected to maximize the coverage of the suggested 47.5° of TPT structural torsion [1] within the T-MTS’s operating limits, thereby allowing the ST measurements to be validated across the potential range of the TPT’s dynamic torsion. Under each testing condition, the dynamic torsion angles of the phantoms were measured separately by both ST and MT methods. All tests were repeated three times.

#### 2.1.3. Acquisition of Phantom Images

To capture the ultrasound images required for ST and MT measurements, a 7.5 MHz, 128-channel, 38.4 mm linear array ultrasound probe (L154BH_T3300, BenQ Medical Technology, Taipei, Taiwan) was placed horizontally at the midsection of the phantom (20 mm away from both clamps) with its position supported by a 3D-printed probe holder (Figure 1B). The center placement of the ultrasound probe ensured that the measured cross-section of the phantom was far from the influence of boundary conditions at the clamping sites, according to Saint-Venant’s principle [41]. Since the phantom was rotated only at the lower and not the upper clamp of the T-MTS, a decreasing gradient of dynamic torsion angles would be generated from the phantom’s bottom to the top. As a result, the dynamic torsion angle measured at the midsection of the phantom would be smaller than the set rotation angle of the T-MTS lower clamp, with its theoretical maximum being 1/2 of the set rotation angle. Hence, the actual dynamic torsion angle at the center of the phantom was measured with MT analysis (refer to Section 2.1.5), which served as the reference value for evaluating ST accuracy.

#### 2.1.4. Speckle-Tracking Analysis

In the ST analysis, radiofrequency data of the phantoms’ cross-section images were recorded at a frame rate of 80 Hz during T-MTS dynamic torsion testing. Three recordings of radiofrequency data were acquired under each testing condition, resulting in a total of nine recordings for each phantom sample. After data acquisition, the recorded radiofrequency data were imported into a custom ST algorithm for offline calculation of the rotation angle ($\theta_{ST}$) of the phantom’s cross section. Since the phantom rotated only at the T-MTS lower clamp but not the upper clamp, the rotation angle ($\theta_{ST}$) at the phantom’s cross section can be regarded as the dynamic torsion angle of the phantom.

The ST algorithm used was based on a previously validated method [33,42], which incorporated a block-matching method to track the movement of the ultrasound speckles and used normalized cross-correlation (NCC) values as the determining parameter for finding the best matches of speckle signals between image frames. The ST analysis was executed with a custom MATLAB algorithm (R2023a, MathWorks, Inc., Natick, MA, USA).

First, the speckle movement (axial and lateral) of the phantom during dynamic torsion testing were analyzed using matching blocks (kernels) of size 7 × 7 pixels (0.4 mm in the axial and 2.1 mm in the lateral direction) within a search area of approximately 260 × 128 pixels (15.0 mm in the axial and 38.4 mm in the lateral direction, corresponding to the size of the recorded radiofrequency data). The axial kernel overlap was 80%, and the lateral kernel shift was 1 pixel. For each kernel, a 2D cross-correlation analysis [42] was conducted to identify the best-matching kernel in the subsequent frame by locating the global peak in the NCC matrix, thereby determining the displacement of the speckle signals. To enable analysis of speckle movement at the sub-pixel level and to suppress the potential influence of peak-hopping artefacts arising from signal decorrelation due to speckle rotation or out-of-plane motion during the cross-correlation analysis, an 8:1 2D spline interpolation was applied to the NCC matrix [42,43]. Furthermore, a sample-based recorrelation analysis proposed by Li et al. [42] was performed to improve the accuracy of the lateral speckle displacement measurements. Subsequently, 2D speckle axial and lateral displacement matrices were generated from cumulative displacements of each kernel through all the pairs of consecutive frames recorded in the radiofrequency data.

Second, a region of interest (ROI) was manually defined according to the boundary of the phantom image on the grayscale image converted from the radiofrequency data using log compression and the Hilbert transform [38]. This defined ROI was then applied to the previously calculated lateral and axial displacement matrices to extract meaningful speckle displacement data for subsequent dynamic torsion analyses.

**Figure 2:**
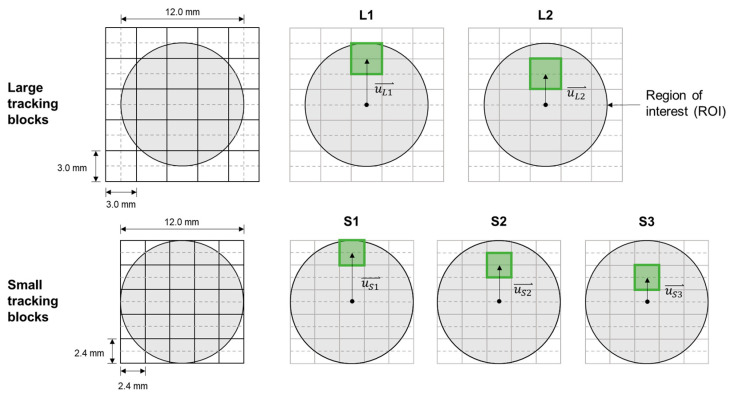
Tracking block configurations used in the speckle-tracking algorithm. Five combinations of tracking block size and position were used: large (L1 and L2) and small (S1, S2, and S3). The sizes of large and small tracking blocks were 1/4 and 1/5 the diameter of the phantom’s cross-section, respectively. The locations of the tracking blocks were 2×, 1.5×, or 1× their size away from the center of the phantom.

Third, a set of tracking blocks was automatically defined within the ROI by the algorithm to serve as virtual markers for tracking the rotational movement of the phantom (Figure 2). Because nonuniform speckle displacements within the ROI are expected under rotational movement, these tracking blocks served the purpose of putting speckles into groups, before individually calculating their averaged accumulative lateral and axial displacements. Two sizes of square-shaped tracking blocks were automatically defined by the custom MATLAB algorithm to study the effect of tracking block size on ST measurement accuracy (Figure 2). The large tracking block was 1/4 phantom diameter in size (3 × 3 mm), and the small tracking block was 1/5 phantom diameter in size (2.4 × 2.4 mm^2^). Furthermore, two different positions of the large tracking blocks (L1 and L2) and three locations of the small tracking blocks (S1, S2, and S3) were also tested to study the effect of tracking block position on ST accuracy (Figure 2). The L1 and L2 tracking blocks were positioned 1.5× and 1× their size away from the center of the phantom, respectively. The S1, S2, and S3 tracking blocks are located 2×, 1.5×, and 1× away from the center of the phantom, respectively.

Finally, net lateral and axial displacements of the tracking blocks relative to the ROI displacement were calculated. As the averaged lateral and axial displacements of the tracking blocks define their tangential displacements relative to the rotation center of the ROI, the dynamic torsion angle ($\theta_{ST}$) of the phantom was then calculated as the angle of the arc that the tracking blocks traveled over time.

The quality of the ST results was assessed using the criteria NCC > 0.9 and coefficient of variance (CV) < 30%, as in previous studies [31,33,44,45]. Here, the NCC was used to assess the estimation accuracy and robustness of the speckle displacement measurements [24,31,33], and the CV was used to assess whether measurements within the tracking blocks varied excessively, thus representing a risk of tracking error [33,45]. Trials that matched the criteria were retained for the final statistical analysis, with 91% of all recorded trials passing the quality criteria.

#### 2.1.5. Marker Tracking Analysis

For MT analysis, grayscale ultrasound images of the phantoms’ cross-section were recorded at a sampling rate of 20 Hz. Before image acquisition, a metal stapler pin was inserted into the center of the phantom to serve as a hyperechoic imaging marker. Three videos of grayscale ultrasound images were recorded under each T-MTS testing condition, and a total of nine videos were recorded for each phantom sample. The recorded videos were analyzed offline using a custom MATLAB algorithm to calculate the real-time dynamic torsion angle ($\theta_{MT}$) of the phantom during the tests. The center of the phantom and the position of the marker were automatically identified in each frame from the recorded grayscale images. Then, $\theta_{MT}$ was calculated by measuring the angle of arc that the marker had traveled during the test (Figure 1C). The acquired $\theta_{MT}$ was considered to be the actual dynamic torsion angle of the phantom during the T-MTS test and was taken as the reference to evaluate ST accuracy.

#### 2.1.6. Statistical Analyses

The validity of assessing dynamic torsion via ST was evaluated using linear regression of the ST results ($\theta_{ST}$) and the benchmark MT results ($\theta_{MT}$) during torsional mechanical tests [33,46,47], as well as descriptive statistics of mean absolute errors (MAEs), calculated using the formula $MAE=\sum \left|{\theta_{ST}-\theta }_{MT}\right|/n$ (where n is the experimental sample size), to present the measurement error of ST relative to the MT method [33,46]. To assess whether the ST measurement error was comparable to the hypothesized 3° reported in previous studies [24,25], one-sample *t*-tests of the MAEs with a test value of 3° were conducted (α = 0.05, two-tailed). In addition, to compare the performance of ST under different T-MTS testing conditions (three levels) and tracking block configurations (five levels; repeated measures), a 3 × 5 two-way repeated-measures analysis of variance (ANOVA) was conducted on MAEs (α = 0.05, two-tailed). In this ANOVA test, if the assumption of sphericity was not met, a Greenhouse–Geisser (GG) correction was applied to the F-statistics, and the adjusted F and *p* values (p_(GG)_) were reported. All statistical tests were conducted using the SPSS software (version 22.0, IBM, Armonk, NY, USA).

The sample size required for the regression analysis (1 predictor) to achieve 80% statistical power with an alpha error probability of 0.05 was calculated using G*Power software (3.1.9.7, Universität Kiel, Kiel, Germany). Based on previously reported coefficient of determination (*R*^2^) values of 0.63–0.99 in the ST validation studies [33,46,47], the minimum required sample size was 8.

### 2.2. In Vivo Application of ST Dynamic Torsion Assessment on Human TPT

#### 2.2.1. Participants

Nineteen healthy participants aged 20–65 years were included in the study. To control for foot posture variability among participants, the study only included participants with neutral feet scoring 0–5 points in the six-item Foot Posture Index (FPI-6) [48,49]. Individuals with pronated (FPI-6 score ≥ 6) or supinated (FPI-6 score < 0) foot postures, a history of surgeries, recent injuries within the past three months, or any neurological or vascular pathologies requiring medical attention in their lower extremities were excluded from the study.

#### 2.2.2. Study Procedure

**Figure 3:**
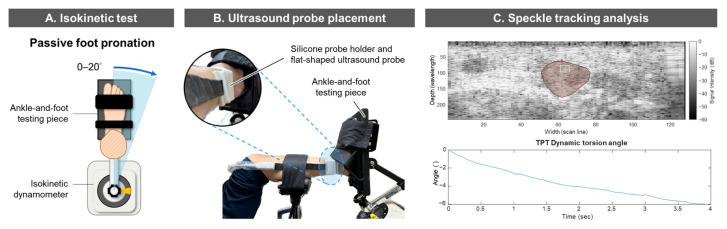
In vivo dynamic torsion assessment of the tibialis posterior tendon (TPT). (**A**) A passive foot pronation test was conducted using an isokinetic dynamometer to induce dynamic torsion of the TPT; (**B**) During the dynamic task, a flat-shaped ultrasound probe was fixated with a silicone probe holder at 1-cm above the medial malleolar to record transverse plane radiofrequency data of the TPT; (**C**) The dynamic tendon torsion angle was calculated offline in the custom MATLAB ST algorithm. The red area in the ultrasound image represents the region of interest (ROI), and the white box represents the tracking block.

The participants were asked to perform a passive foot pronation dynamic motion task on an isokinetic dynamometer (System 4, Biodex Medical Systems, Inc., Shirley, NY, USA) (Figure 3A) to induce dynamic torsion of the TPT. Participants were asked to sit on the isokinetic dynamometer, with their tested leg fixed horizontally and the foot in firm contact with the ankle-and-foot testing piece of the dynamometer. Participants were then instructed to fully relax their lower extremities to allow the isokinetic dynamometer to carry their foot passively from 0° to 20° of foot pronation at a constant rotational velocity of 5° per second controlled by the computer. Passive foot pronation was performed using the same isokinetic testing protocol (passive mode, ankle eversion/inversion pattern, eccentric/eccentric contraction, and 1-hard cushion setting), ensuring consistent movement across trials and participants. This combined movement mainly consists of external rotation (abduction) in the transverse plane and eversion in the frontal plane [50]. The 0–20° foot pronation range and the 5° per second rotational velocity were selected based on previous isokinetic testing protocols [51,52] and to ensure completion within 4 s, enabling the ultrasound radiofrequency recording (described in Section 2.2.3) to cover the entire duration of the passive foot pronation motion. Three to five practice trials were performed to familiarize the participants with the testing procedure. Then, three repetitions of the passive foot pronation test were conducted.

#### 2.2.3. Acquisition of Tendon Images

To record the dynamic torsion of the TPT during the passive foot pronation test, a custom-made flat-shaped ultrasound probe (7.5 MHz, 128-channel, 38.4 mm; L124BH_T3300, BenQ Medical Technology, Taipei, Taiwan) with the same technical specifications as those of the L154BH_T3300 was fixed with a custom silicone probe holder at 1 cm proximal to the medial malleolus, along the TPT’s short axis. Radiofrequency data were collected at a sampling rate of 80 Hz for ST analysis (Figure 3B). A custom-made silicone probe holder was used to fix the probe to the participants’ limb and stabilize the probe during the dynamic task. Three 5-s recordings of radiofrequency data were taken during the passive foot pronation test.

#### 2.2.4. Speckle-Tracking Analysis of TPT Dynamic Torsion

The calculation of dynamic TPT torsion angles was performed offline using the same MATLAB custom ST algorithm reported in the first part of the study. The ROI was manually selected according to the boundary of the TPT’s cross-section on the converted grayscale image from the radiofrequency data (Figure 3C). Five tracking blocks with different sizes and locations (L1, L2, S1, S2, and S3) were automatically defined based on the dimensions of the ROI. The net lateral and axial displacements of these tracking blocks relative to the ROI were first calculated. Then, the dynamic torsion angle (θ) of the TPT’s cross-section during the passive foot pronation test was calculated by the tangential displacement acquired from the tracking blocks relative to the center of the ROI. A positive angle indicates internal dynamic torsion of the tendon, and a negative angle indicates an external dynamic torsion of the tendon.

Quality checks were again performed for the ST results using the criteria of NCC > 0.9 and CV < 30%. Trials that met the set criteria were retained for the statistical analyses, with 93% of the trials qualifying.

#### 2.2.5. Collection of Foot Pronation Angles

Real-time data of the foot pronation angle during the passive foot pronation test were recorded using a data collection system (Noraxon TeleMyo Mini Receiver and MyoResearch 3.8 software, Noraxon USA, Scottsdale, AZ, USA) with the isokinetic dynamometer at a sampling rate of 1500 Hz, for comparison with the ST-measured dynamic torsion angles of the TPT. The synchronization of ST-measured angles and foot pronation angles was performed offline using a custom MATLAB script, with the time series of the two datasets matched at a frame rate of 20 Hz.

#### 2.2.6. Reliability Test

Test–retest reliability (inter-session) of the in vivo ST analysis on TPT dynamic torsion was assessed in 10 participants, with an interval of at least 1 week between testing sessions. In each testing session, the ST measurements were repeated three times. To ensure consistency in the testing protocol and control operator errors, ultrasound imaging was performed by the same operator (7 years of experience), and probe positioning was guided by bony landmarks (1 cm proximal to the medial malleolus) and tape measures, then fixed with a silicone probe holder.

#### 2.2.7. Statistical Analyses

The performance of ST analysis in TPT dynamic torsion assessment was evaluated by the coefficient of determination (*R*^2^) between the ST-measured dynamic torsion angles and foot pronation angles. To investigate whether different tracking block configurations (five levels, repeated measures) affect the ST-measured dynamic torsion angle of the TPT, a 1 × 5 one-way repeated measures ANOVA was conducted. A Greenhouse–Geisser (GG) correction was applied if the assumption of sphericity was not met. In addition, to evaluate test–retest reliability, the interclass correlation coefficient (ICC [3, 3]) was used to assess relative reliability, and the standard error of measurement (SEM) and minimal detectable change (MDC) were used to assess absolute reliability. Bland–Altman plots were also analyzed to identify systematic and proportional biases between inter-session measurements. The significance level of all statistical tests was set at α = 0.05 (two-tailed). All statistical tests were conducted using the SPSS software (version 22.0).

The sample size required for the one-way repeated measures ANOVA to achieve 80% statistical power with an alpha error probability of 0.05 was calculated using G*Power software (3.1.9.7) based on the statistical result from the first part of the study. The effect size (η^2^) of the tracking block effect on ST results was 0.100. Therefore, the required sample size was calculated to be at least 12.

## 3. Results

### 3.1. Validation of ST Dynamic Torsion Assessment

**Figure 4:**
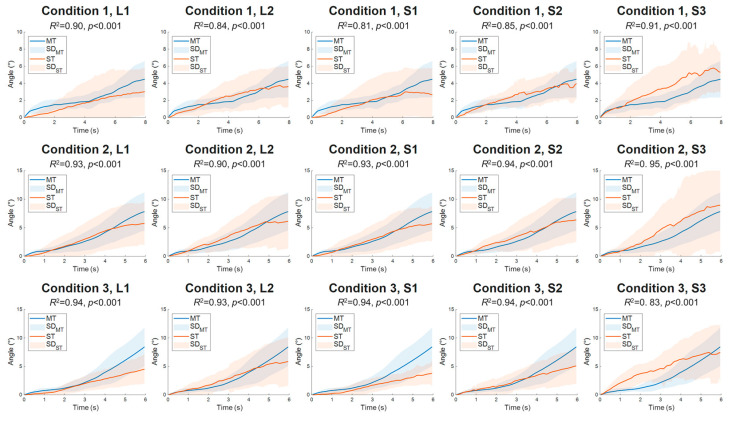
Results of dynamic torsion angle measurements from speckle tracking (ST) (orange lines) and marker tracking (MT) results (blue lines) during the torsional mechanical tests under three T-MTS testing conditions and five tracking block configurations. In each graph, the blue and orange lines denote the average dynamic torsion angle trajectory over time of the 10 silicone phantom samples, measured by the MT and ST methods, respectively. The blue- and orange-shaded areas depict the standard errors of the measurements analyzed at every frame of the ultrasound image recording. The coefficient of determination (*R*^2^) and *p*-value for comparisons between ST and MT measurements are provided for each combination of testing conditions and tracking block configurations.

Ten silicon phantoms were tested using the T-MTS to assess the accuracy of ST in measuring dynamic torsion angles, compared against MT results. Significant correlation coefficients were noted between time series ST and MT data across all T-MTS testing conditions and tracking block configurations (*R*^2^ = 0.81–0.95, *p* < 0.001) (Figure 4). The MT results ($\theta_{MT}$) under testing conditions 1, 2, and 3 were 4.2 ± 2.1°, 7.8 ± 3.4°, and 8.4 ± 3.4°, respectively. The MAEs of ST results compared to MT results were 2.2–5.8° under all combinations of testing conditions and tracking block configurations (Table 1); most of them were similar to the hypothesized 3° (*p* > 0.05, d ≤ 0.65), except under testing condition 3, where the S3 tracking block showed a significantly larger MAE than 3° (*p* = 0.045, d = 0.74).

In two-way repeated measures ANOVA (Table 1), MAEs showed no significant interaction effect (F(2.4, 21.6) = 0.329, p_(GG)_ = 0.761, η^2^ = 0.035) or tracking block effect (F(1.8, 16.5) = 0.999, p_(GG)_ = 0.383, η^2^ = 0.100). Despite a significant overall effect of T-MTS testing conditions observed for MAE values (F(2, 18) = 92.601, *p* = 0.022, η^2^ = 0.346), post hoc pairwise comparisons with Bonferroni correction showed no significant differences in MAEs between each pair of the T-MTS testing conditions (*p* ≥ 0.058, d ≤ 0.90).

### 3.2. In Vivo Application of ST Dynamic Torsion Assessment on Human TPT

**Figure 5:**
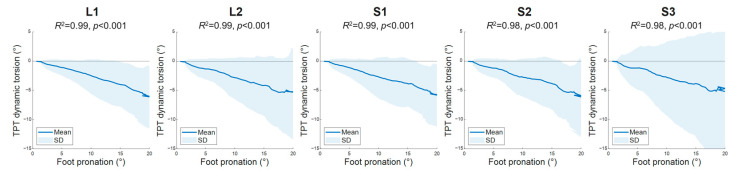
Correlation between speckle-tracking results and foot pronation angle under five tracking block configurations. In the graph, the line denotes the mean dynamic torsion angle of the tibialis posterior tendon (TPT) in the 19 participants during passive foot pronation. The blue-shaded area depicts the standard deviations of the measurements at each frame during the ultrasound radiofrequency data recording. The coefficient of determination (*R*^2^) and *p*-values for comparisons between the TPT dynamic torsion angle and the foot pronation angle are provided for each tracking block configuration.

Nineteen healthy participants (age = 29.5 ± 10.3 years; height = 165 ± 10 cm; weight = 59 ± 10 kg; median FPI-6 score = 3) were included in the study to evaluate the applicability of ST dynamic torsion assessment in vivo. During passive foot pronation, ST-measured dynamic torsion angles ranged between −4.4° and −5.5° (Table 2). These results were correlated with the passive foot pronation angle, where the coefficients of determination (*R*^2^) were 0.98–0.99 across all tracking block configurations (*p* < 0.001) (Figure 5). The result of the one-way repeated measures ANOVA showed no significant effect of tracking block configuration on the ST-measured dynamic torsion angles (F(1.2, 19.8) = 0.189, p_(GG)_ = 0.705, η^2^ = 0.011) (Table 2).

**Figure 6:**
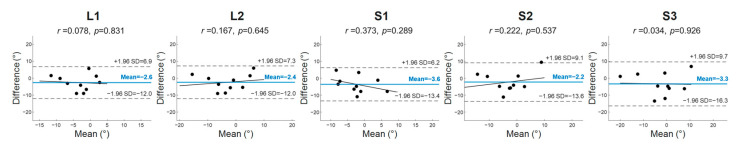
Bland–Altman plots evaluating in vivo speckle-tracking test–retest reliability across five tracking block configurations. The scatter dots denote the means and differences in each pair of intra-session measurements, the solid horizontal lines denote the mean differences between the test and retest measurements, the dashed horizontal lines denote the upper and lower limits of agreement (±1.96 times the standard deviation of the measurement differences), and the black lines denote the regression fit of the differences on the means. The systemic biases of the measurements were 2.2–3.6° across all tracking block configurations. No proportional biases were shown by correlation analyses between mean and differences in measurement values (*p* > 0.05). The correlation coefficients and *p*-values for the relationship between the mean difference in test and retest measurements (vertical axis) and the mean magnitude of measurements (horizontal axis) are provided for each tracking block configuration.

Ten participants completed the test–retest procedure (Table 3). For the passive foot pronation task, the range of ICC(3,3) values was 0.73–0.87, the SEM was 1.8–2.6°, and the MDC was 5.0–7.1°. For the isometric foot supination task, the range of the ICC(3,3) values was 0.72–0.95, the SEM was 0.7–3.2°, and the MDC was 1.9–8.9°. Analysis of the Bland–Altman plots (Figure 6) showed that the bias of the measurements lay between 2.2° and 3.6° across all testing conditions and tracking block configurations. No significant proportional biases were noted through correlation analyses between test–retest differences or means across all testing conditions (*p* > 0.05, |*r*| ≤ 0.373).

## 4. Discussion

### 4.1. Validation of ST Dynamic Torsion Assessment

This is the first study to utilize ultrasound ST to assess dynamic torsion angles. Ten silicone phantoms were tested on the T-MTS, with ST showing accurate dynamic torsion angle measurements with correlation coefficients of *R*^2^ = 0.81–0.95 (Figure 4) and MAEs of 2.2–5.8° (Table 1) across all tested ranges of dynamic torsion (testing conditions) and tracking block configurations. Supported by the strong correlation between ST and MT measurements (*p* < 0.001), and the generally insignificant differences between MAEs of the ST measurements and the hypothesized 3° criterion (*p* ≥ 0.045, d ≤ 0.074), the current study suggests that ST is capable of detecting transverse plane rotation of the phantom and measuring dynamic torsion angles with acceptable accuracy.

The strong correlation and the small errors of ST measurements compared to the reference methods are consistent with the findings of previous ST validation studies. These reports have measured longitudinal strain or displacement of phantoms and musculoskeletal tissues, and are summarized in Table 4. The reported correlation coefficients between ST and reference methodologies ranged from 0.63 to 0.99 [33,38,46,47,53], with measurement error metrics in displacements (including root mean square error, mean error, and MAE) ranging from 0.05 to 1.90 mm and strain error ranging from 0.21% to 14.85% [24,25,33,35,46,47,53,54]. In the current study, the correlation coefficients were 0.81–0.95 across all testing conditions and tracking block configurations in the ST algorithm, comparable with previously reported ranges. The MAEs in this study, while analyzed in degrees (Table 1), can be estimated as their equivalent errors in millimeters ($E_{mm}$) in tangential displacements of the tracking blocks for the convenience of comparison. With the estimation formula of $E_{mm}=2\pi r\times \left(MAE/360{}^{\circ}\right)$, where r is the distance of the tracking block to the center of the phantom (Figure 2), the equivalent measurement errors of the ST results were 0.12–0.42 mm, also comparable to the reported MAE ranges of 0.15–1.90 mm in the literature [33,46,54]. Although the S3 tracking block under testing condition 3 showed significantly larger MAE compared to the hypothesized 3° measurement error (*p* = 0.045, d = 0.74; Table 1), its 95% confidence interval of 3.1–8.1°, converted to 0.12–0.34 mm, is also comparable to the literature-reported MAE ranges [33,46,54]. As a result, this study believes that the current transverse plane ST analysis has similar performance to previous validated longitudinal plane ST methodologies. The presented ST technique can therefore be regarded as an accurate method for measuring dynamic torsion angles.

To evaluate of ST accuracy over the potential range of in vivo TPT dynamic torsion, the T-MTS testing conditions were selected to approximate the reported 47.5° of TPT structural torsion, including 20°, 30°, and 40° of rotation at the bottom end of the phantom. Under these testing conditions, the dynamic torsion angles of the phantoms ranged from 4.2 ° to 8.4° at their vertical centers. This range of phantom dynamic torsion angles is consistent with the previously reported 4.36° Achilles tendon dynamic torsion in vivo during isometric plantar flexion [17], as well as the 4.4–5.5° in vivo TPT dynamic torsion angles found in the second part of the study (Table 2), suggesting that the current T-MTS testing conditions for ST validation may be suitable for assessing physiological dynamic tendon torsion in vivo. In the evaluation of ST accuracy under these T-MTS testing conditions, the marginal means of MAEs were 2.6 ± 0.8° for condition 1 (under 4.2 ± 2.1° of dynamic torsion), 4.3 ± 3.5° for condition 2 (7.8 ± 3.4° of dynamic torsion), and 5.3 ± 3.3° for condition 3 (8.4 ± 3.4° of dynamic torsion). Despite no statistically significant effects of testing conditions on these MAEs were observed (*p* > 0.058), a larger effect size (d = 0.90) was observed in the pairwise comparison of the marginal means of MAEs for conditions 1 and 3 (*p* = 0.058, power = 71.7%). This trend of larger MAEs in condition 3 than in condition 1 may be attributed to the greater magnitude of phantom rotation and elongation during T-MTS testing, which increases the risk of speckle rotation and out-of-plane motion, respectively. According to the literature, both of these motions may contribute to speckle signal decorrelation and reduce the accuracy of ST measurements [42,43]. Despite the non-significant pairwise comparison between conditions 1 and 3, there is a 28.3% chance of a type II statistical error, suggesting that the ST accuracy in condition 3 may be poorer than in condition 1. Nevertheless, since the magnitudes of MAEs were similar to the previously reported error levels in established longitudinal ST methodologies, the study suggested that the ST has acceptable accuracy within the current tested dynamic torsion range (approximately 4–8°). However, it should be noted that there may be a tendency toward larger measurement errors as the dynamic torsion range increases.

In the design of this validation experiment, potential sources of ST errors suggested in the literature [44] were controlled for. These controls included establishing a quality control plan before data collection for selecting high quality results (NCC > 0.9 and CV < 30%), recording radiofrequency data instead of B-mode image, ensuring speckle dimensions were smaller than pixel resolution (using 40–70 µm glass bead powder to create speckles), using a sufficient frame rate to capture speckle motion (80 Hz), and accounting for accumulated subpixel displacements in the ST algorithm [42,44]. However, the risk of out-of-plane motion may be present in the study design due to the simultaneous twist and pull of phantoms on the T-MTS. The T-MTS was purposely programmed to twist and pull the phantom to realistically recreate the complex motions that occur in in vivo tendons [1,18,26]. Although the pulling motion of the phantom can cause elevational displacement of the ultrasound probe (displacement perpendicular to the long axis of the probe), this displacement at the midsection of the phantom (≈5.6/2 = 2.8 mm in condition 3) was covered by the tested slice thickness of 3.22 ± 0.21 mm (sample size = 10) of the ultrasound probe used by the current study [56]. As a result, a portion of the original speckle patterns at the start of the radiofrequency data recording was retained in the image frame at the end of the test. In addition, the correlations of ST and MT were strong in condition 3, and no significant differences in the MAEs of ST were noted in the pairwise comparisons between testing conditions (*p* > 0.05). Therefore, the result may imply that ST could tolerate some extent of out-of-plane motion, as long as the elevational displacement does not exceed the slice thickness of the ultrasound probe.

### 4.2. In Vivo Application of ST Dynamic Torsion Assessment on Human TPT

The second part of the study aimed to apply the validated ST dynamic torsion assessment on in vivo TPT to evaluate the feasibility and reliability of TPT dynamic torsion measurements. The strong correlation between the ST measurements and passive foot pronation angles (*R*^2^ = 0.98–0.99; Figure 5), and the similar measured values of TPT dynamic torsion angles from all tracking block configurations (p_(GG)_ = 0.705; Table 2), demonstrated the capability of the ST algorithm to measure changes in in vivo TPT torsion angles in response to an increase in passive foot pronation angle. In addition, all ST measurements showed moderate to good test–retest reliability (ICC = 0.73–0.87), close to previously reported ICC ranges of 0.72–0.99 in measuring in vivo longitudinal Achilles tendons displacement and strain during passive ankle dorsi–plantar–flexion movements [30,57]. These findings support the study’s hypothesis that ST is a feasible and reliable method for measuring in vivo dynamic torsion of the TPT.

**Figure 7:**
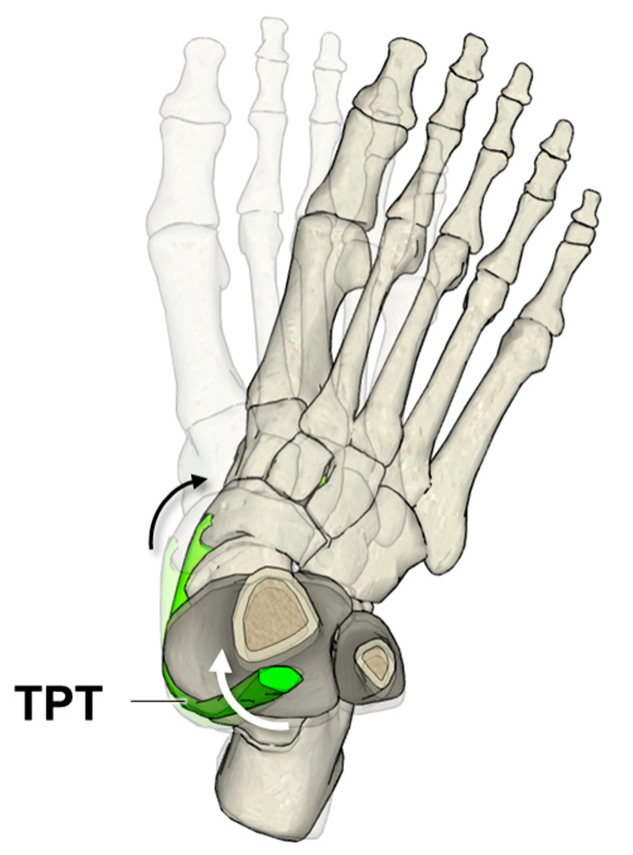
Illustration of the suggested tibialis posterior tendon (TPT, highlighted in green) external dynamic torsion (white arrow) during foot pronation (black arrow). (Source of 3D models: BodyParts3D (Data version 4.3), © The Database Center for Life Science licensed under CC Attribution-Share Alike 2.1 Japan.).

This study observed negative dynamic torsion angles of the TPT with all tracking block configurations during foot pronation (Table 2), indicating external dynamic torsion of the TPT. This is consistent with the suggested “unwinding” movement on the structurally internally twisted TPT during foot pronation [1]. As reported in the literature, the pronation of the foot involves tri-planar joint movements, which primarily consist of external rotation (abduction) in the transverse plane and eversion in the frontal plane [50]. In previous studies of joint kinematics, 20° of foot external rotation (abduction) caused 6.1° of talar bone external rotation relative to the tibia and fibula bones in the talocrural joint in the transverse plane [58]; meanwhile, 20° of foot eversion was found to cause 7.5° of navicular bone supination relative to the talar bone in the talonavicular joint in the frontal plane [59]. Collectively, these joint motions could contribute to external torsion of the TPT, since its anatomical course lies between the navicular tuberosity and the proximal tibia and fibula bones (as illustrated in Figure 7). Given the testing condition of 20–foot pronation on the isokinetic dynamometer, the current study believes the finding of dynamic external torsion of the TPT during foot pronation is reasonable.

Moreover, implied from the linear relationship between the TPT dynamic torsion angle and the foot pronation angle (*R*^2^ > 0.98), the external dynamic torsion may be a mechanical response (behavior) of the TPT closely related to the pronation movement of the foot. Since the ST-measured external dynamic torsion of the TPT indicates the change in its torsion angle during pronation, it can be inferred that the inherent structural torsional characteristics of the TPT would change under foot motion. According to previous studies [13,15], a twisted tendon is stiffer and better able to endure greater tensile stress and strain before failure compared with a straight tendon; therefore, if the observed external dynamic torsion of the TPT unwinds the anatomically internal-twisted TPT structure [1], the mechanical properties (e.g., stiffness and failure stress or strain) of the TPT under tensile stress may become weaker with increasing foot pronation. Although this hypothesis requires further examination before a conclusion can be drawn, it may be clinically relevant, as the TPT of individuals with overpronated foot posture may be unwound or straightened by foot pronation, thereby increasing the risk of tendon injury due to its compromised mechanical properties. Thus, verifying the relationship between pronation and TPT mechanical properties might facilitate understanding of the potential mechanism underlying TPT tendinopathy (i.e., tenosynovitis or tendinosis) in clinical conditions such as overuse sports injury or posterior tibial tendon dysfunction in patients with flatfoot [60,61,62].

**Figure 8:**
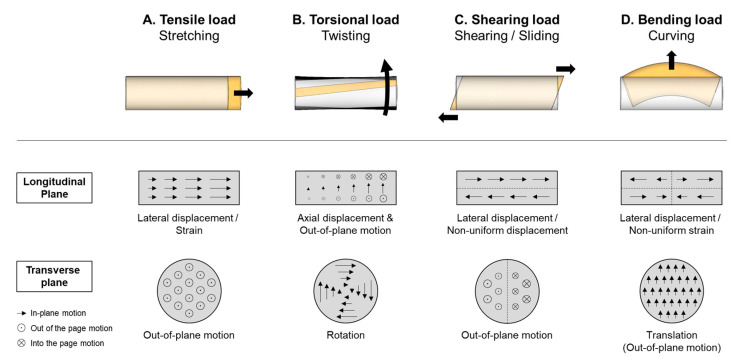
Illustration of the potential mechanical loadings applied to the tendon during joint movement. During normal joint movement, tendons may be subjected to (**A**) tensile, (**B**) torsional, (**C**) shearing, and (**D**) bending loads, resulting in stretching, twisting, shearing, or curving deformations, respectively. Examples of the deformed tendons are presented in yellow. (**A**) Under tensile load, lateral displacement or strain can be observed from the longitudinal plane. (**B**) Under torsional load, rotation of the tendon’s cross-section can be observed from the transverse plane. (**C**) Under shearing load, intratendinous sliding or non-uniform displacements can be observed from the longitudinal plane. (**D**) Under bending load, stretching and compressive strain can be observed from the longitudinal plane, and a translation motion (potentially combined with an out-of-plane motion) can be observed from the transverse plane.

Combining the findings from the evaluation of ST validity in its first part, and the assessment of feasibility and reliability in its second part (in vivo TPT dynamic torsion), this study demonstrated that the transverse plane ST algorithm is valid and can reliably observe the external dynamic torsion of the TPT in vivo during passive foot pronation. The moderate to good reliability (ICC = 0.73–0.87) and strong correlation (*R*^2^ > 0.98) between ST dynamic torsion measurements and joint rotation angles further suggest the potential use of transverse-plane ST to assess the physiological or pathological mechanical behaviors of the tendon during dynamic movement. During joint movement, a tendon may be subjected to mechanical loading in multiple directions, including tensile, torsional, shearing, and bending loads, resulting in complex stretching, twisting, shearing, and curving deformations (Figure 8) [14,63]. Under the premise that tendon injuries or tendinopathy may arise from prolonged or repetitive overloading or unloading [64,65,66,67,68], investigating the torsional behavior of a tendon using transverse plane ST may provide a more comprehensive understanding of the underlying mechanical mechanisms in the pathogenesis of tendinopathy [66,69]. Like the assessment of tensile overloading or unloading using longitudinal tendon displacement or tensile strain [70,71], the assessment of dynamic torsion could also provide insights into the concepts of “torsional overloading” or “torsional unloading,” each reflected by an increase or decrease in the magnitude of dynamic tendon torsion. For example, studies have suggested that excessive torsion may constrict the vascular supply to tendon tissue, thereby increasing the risk of tendinopathy [1,5,10,11]. In this case, an increase in dynamic torsion of the affected tendon may be observed if compared to a healthy one. Therefore, using ST to observe differences in dynamic torsion between pathologic and healthy tendons, or changes with tendon pathology or intervention, may be valuable for clinical assessments of tendinopathy. Future studies may continue to develop the ST dynamic torsion assessment methodology and explore its functional and clinical relevance.

In designing the in vivo application experiment, the same error and quality control plans were followed as those conducted in the first part of the study. To eliminate the risk of operator-induced out-of-plane motions, caused by the dynamic motions of the limb when performing ST assessments [44], a flat-shaped ultrasound probe combined with a silicone-made probe holder was included to fit the contour of the participants’ ankles; this ensured firm fixation of the probe to the skin in this part of the study (Figure 3B). Moreover, to control the potential influence of foot postures on the characterization of the dynamic torsion of TPT, the study only included participants who scored 0–5 on the FPI-6. Nevertheless, the study has some limitations. First, the passive foot pronation range was fixed at 0–20° for all participants to allow comparison of their ST results across different foot pronation angles. Therefore, the tested range of pronation may not reflect each participant’s functional range of motion. Second, the acquired validation results on silicone phantoms may not reflect the ST performance on in vivo TPT dynamic torsion measurements. Third, due to the lack of ground-truth data for the in vivo measurements, the accuracy of ST-measured dynamic torsion angles was not evaluated. Consequently, the in vivo ST measurements may not accurately reflect the actual magnitude of dynamic torsion of the TPT, but rather a set of unique estimated values obtained under the testing conditions examined in this study. Future cadaveric studies [47] or, if possible, intraoperative measurements [25,54] may be warranted to obtain ground-truth data on dynamic torsion of the TPT. Finally, because this study analyzed only in-plane speckle displacements in the TPT’s cross-section image, there may be a risk of out-of-plane speckle motion due to longitudinal tendon excursion or shearing deformation [7,40] (Figure 8). The out-of-plane motion could induce speckle signal decorrelation and incorrect matching of speckle signals between consecutive frames, potentially leading to underestimation of the speckle displacements [42,43,72]. Although the risk of out-of-plane motion might be reduced if the ultrasound probe’s slice thickness encompasses the magnitude of the longitudinal deformations (in which the original speckle pattern remains in frame), its impact on ST measurements may be inevitable. Therefore, caution is warranted when interpreting the magnitude of ST measurements. A more robust control of out-of-plane motion may require conducting biplanar ST analysis or implementing 3D ST technology to include a comprehensive analysis of the complex tendon motion.

### 4.3. Suggestions for Future In Vivo ST Dynamic Torsion Assessment

The current study applied the ST algorithm in vivo to measure the dynamic torsion angle of the TPT. In future studies, the ST algorithm may be applied to assess in vivo dynamic torsion of various tendons under different movement patterns (e.g., isometric or isotonic contractions). Therefore, further refinements to the ST algorithm and testing protocol would be necessary to adapt to varying testing conditions and to improve tracking accuracy and measurement reliability.

For the ST algorithm, optimization of ST parameters, including kernel sizes and search area dimensions, can be performed before applying ST analyses to identify the best-performing settings for the specific magnitude and orientation of the frame-to-frame speckle movements, thereby reducing signal decorrelation and improving tracking quality [42,47,53]. Additionally, the ST algorithm can average speckle displacements from multiple tracking blocks, similar to segment analysis in ST echocardiography [20,22,23], to reduce the influence of local noise on dynamic torsion measurements.

For the testing protocol, more repetitions per session (e.g., five repetitions) could be performed to stabilize session means, thereby reducing intra-session measurement variability and improving reliability. In addition, a flat-shaped ultrasound probe would be preferable for dynamic testing to improve probe stabilization and reduce motion artifacts [73,74]. The positioning and orientation of the ultrasound probe relative to the body during movements could also be monitored using a 3D motion-capture system with attached reflective markers [74,75]. Finally, ST analyses of multiple cross-sections of the same tendon can be used to observe the dynamic torsional characteristics of tendons at different anatomical positions and to evaluate regional variability in their dynamic torsion angles [17].

## 5. Conclusions

The present study is the first to validate the use of the transverse plane ST for measuring dynamic torsion movement and apply it to the assessment of TPT dynamic torsion during ankle and foot movement. The results indicate that ST can provide accurate dynamic torsion angle measurements and is reliable for measuring in vivo TPT dynamic torsion characteristics. Future studies could use the validated transverse plane ST methodology to expand the evidence on the functional and clinical relevance of tendon dynamic torsion characteristics.

## Figures and Tables

**Table 1:** Validation of ST dynamic torsion assessment.

	T-MTS Testing Conditions	Tracking Block Configurations				
		L1	L2	S1	S2	S3
ST results (°)	1	3.5 (2.6)	4.0 (2.1)	2.7 (3.1)	3.8 (1.7)	6.1 (3.2)
	2	5.5 (4.0)	6.1 (4.8)	5.6 (3.0)	6.4 (3.7)	8.6 (8.5)
	3	4.6 (3.1)	7.1 (5.0)	3.6 (3.5)	5.3 (3.5)	7.6 (7.0)
MAE (°) [95% CI]	1	2.4 (1.4) [1.4, 3.4]	2.2 (1.2) [1.3, 3.0]	3.4 (1.7) [2.2, 4.6]	2.2 (1.7) [1.0, 3.4]	3.0 (2.2) [1.4, 4.6]
	2	4.3 (4.1) [1.4, 7.2]	4.4 (4.1) [1.4, 7.3]	3.7 (4.1) [0.8, 6.6]	3.8 (3.5) [1.3, 6.3]	5.1 (6.1) [0.7, 9.5]
	3	5.3 (3.9) [2.5, 8.1]	5.8 (4.2) [2.8, 8.8]	5.4 (3.9) [2.6, 8.2]	4.7 (3.2) [2.4, 7.0]	5.6 (3.5) * [3.1, 8.1]
NCC	1	0.99 (0.01)	0.99 (0.01)	0.99 (0.01)	0.99 (0.01)	0.99 (0.01)
	2	0.97 (0.01)	0.98 (0.01)	0.97 (0.01)	0.98 (0.01)	0.98 (0.01)
	3	0.98 (0.01)	0.99 (0.01)	0.98 (0.01)	0.98 (0.01)	0.99 (0.01)
CV (%)	1	4.7 (2.1)	5.2 (2.2)	3.5 (2.3)	3.7 (1.6)	3.7 (1.2)
	2	5.3 (2.5)	5.2 (2.4)	3.9 (1.8)	4.1 (1.5)	3.6 (1.7)
	3	3.7 (1.1)	4.8 (2.0)	3.7 (2.4)	3.8 (1.1)	3.6 (1.8)

Data are presented as mean (standard deviation). The 95% confidence intervals (CIs) for the MAEs are presented in brackets. * Significant difference between MAE and the hypothesized 3° of measurement error.

**Table 2:** In vivo application of ST dynamic torsion angle assessment.

	Tracking Block Configurations					*p*
	L1	L2	S1	S2	S3	
ST results (°)	−5.5 (4.7)	−5.1 (7.0)	−5.4 (4.7)	−5.5 (5.9)	−4.4 (11.5)	0.705 ^a^
NCC	0.99 (0.01)	0.99 (0.01)	0.99 (0.01)	0.99 (0.01)	0.99 (0.01)	-
CV (%)	3.4 (3.4)	3.1 (2.4)	3.5 (3.0)	3.0 (2.3)	2.5 (2.1)	-

Data are presented as mean (standard deviation). ^a^ The F-statistics and *p*-value were adjusted with Greenhouse–Geisser correction.

**Table 3:** Test–retest reliability of in vivo speckle-tracking measurements.

	Tracking Block Configurations				
	L1	L2	S1	S2	S3
ICC(3,3)	0.73 (−0.10, 0.93)	0.87 (0.46, 0.97)	0.74 (−0.06, 0.93)	0.81 (0.22, 0.95)	0.86 (0.44, 0.97)
SEM (°)	2.5	1.8	2.6	2.6	2.5
MDC (°)	7.0	5.0	7.1	7.1	6.8

ICC values are presented with 95% confidence intervals in parentheses.

**Table 4:** Findings of previous speckle-tracking validation studies on phantoms and musculoskeletal tissues.

Reference	Tested Materials/Tissue	Referencing Method	Correlation	Error
Korstanje et al., 2010 [24]	Porcine flexor tendon	MT	-	0.08 mm ^a^
	Human cadaveric FDS tendon	MT	-	0.05 mm ^a^
	Human FDS tendon (in vivo)	MT	-	0.30 mm ^a^
van Doesburg et al., 2012 [25]	Human FDS tendon (in vivo)	MT	-	0.30 mm ^b^
Stegman et al., 2014 [54]	Human FDS tendon (in vivo)	MT	-	0.67–1.90 mm ^c^
Slane et al., 2014 [38]	Polyvinyl chloride-plastisol phantom	DIC	0.99	-
	Porcine flexor tendon	DIC	0.76–0.97	-
Fröberg et al., 2016 [55]	Polyvinyl alcohol (PVA) phantom	MTS	-	0.21% (strain)
	Porcine flexor tendon	MTS	-	0.96–14.85% (strain)
	Human Achilles tendon allograft	MTS	-	0.72% (strain)
Bandaru et al., 2019 [35]	Human cadaveric FDS tendon	MT	-	0.7–1.5 mm **^b^**
Dandois et al., 2021 [47]	Human cadaveric knee MCL	DIC	0.63–0.99	0.26–0.38% (strain)
	Human cadaveric knee LCL	DIC	0.97–0.99	0.24–0.57% (strain)
Wang et al., 2023 [33]	Porcine flexor tendon	MTS	0.98	0.15 mm **^c^**
Kuder et al., 2024 [53]	Porcine knee MCL	DIC	0.99	0.59% (strain)
	Human cadaveric knee MCL & POL	MA	0.91–0.94	-
Chou et al., 2025 [46]	Porcine leg muscle	MTS	0.99	0.8 mm **^c^**

Acronyms: FDS, flexor digitorum superficialis; MCL, medial collateral ligament; LCL, lateral collateral ligament; POL, posterior oblique ligament; MT, marker tracking; DIC, digital image correlation; MTS, mechanical testing system; MA, motion analysis. ^a^ Root mean squared error; ^b^ mean error; ^c^ mean absolute error.

## Data Availability

The raw data supporting the conclusions of this article will be made available by the authors on request.

## Abbreviations

The following abbreviations are used in this manuscript:

ANOVA	Analysis of variance
CI	Confidence interval
CV	Coefficient of variance
FPI-6	Six-item Foot Posture Index
GG	Greenhouse–Geisser correction
ICC	Interclass correlation coefficient
MAE	Mean absolute error
MT	Marker tracking
NCC	Normalized cross-correlation
ROI	Region of interest
SEM	Standard error of the measurement
ST	Speckle tracking
T-MTS	Torsional mechanical testing system
TPT	Tibialis posterior tendon
